# Appetitive responses toward smoking‐related stimuli in abstinence‐motivated, non‐deprived individuals with chronic tobacco dependence: A multi‐methodological investigation

**DOI:** 10.1111/add.70283

**Published:** 2025-12-14

**Authors:** Franziska Motka, Haoye Tan, Seth M. Levine, Sabine Vollstädt‐Klein, Sarah K. Danböck, Katja Bertsch, Markus H. Winkler, Charlotte E. Wittekind

**Affiliations:** ^1^ Division of Clinical Psychology and Psychotherapy, Department of Psychology LMU Munich Munich Germany; ^2^ NeuroImaging Core Unit Munich (NICUM) University Hospital, LMU Munich Munich Germany; ^3^ Department of Addictive Behavior and Addiction Medicine, Central Institute of Mental Health, Medical Faculty Mannheim Heidelberg University Mannheim Germany; ^4^ Mannheim Center of Translational Neurosciences (MCTN), Medical Faculty of Mannheim University of Heidelberg Mannheim Germany; ^5^ German Center of Mental Health (DZPG), Partner Site Mannheim‐Heidelberg‐Ulm Germany; ^6^ Department of Psychology, School of Social Sciences University of Mannheim Mannheim Germany; ^7^ Department of Psychology I (Biological Psychology, Clinical Psychology and Psychotherapy) University of Würzburg Würzburg Germany; ^8^ German Center for Mental Health (DZPG), Partner Site Munich Germany

**Keywords:** addiction, approach bias, EMG, fMRI, smoking, tobacco

## Abstract

**Background and aims:**

Appetitive responses, such as approach biases, are thought to play a crucial role in smoking. This study aimed to compare responses toward smoking‐related stimuli with responses in control conditions (e.g. non‐approach or neutral stimuli) using a multi‐method approach. By examining associations between response measures and with smoking‐related variables, the study sought to extend understanding of their role in abstinence‐motivated, non‐deprived individuals with chronic tobacco dependence.

**Design and setting:**

Cross‐sectional study conducted at a university laboratory and magnetic resonance imaging (MRI) scanner in Munich, Germany.

**Participants:**

362 chronically smoking individuals (51.38% female; data collection: November 2019–March 2023) with moderate‐to‐severe tobacco dependence, enrolled in a smoking cessation study, allowed ad libitum smoking prior to assessment.

**Measurements:**

Responses toward smoking‐related stimuli were assessed using cognitive‐behavioral (reaction‐time‐based approach biases), psychophysiological (electromyography: corrugator supercilii, zygomaticus major and orbicularis oculi for acoustic startle reflex) and neural (functional MRI: regions relevant to smoking cue‐reactivity) measures. Smoking‐related variables were cigarettes per day, tobacco dependence severity and craving. Split‐half reliabilities were estimated for all measures.

**Findings:**

Participants exhibited a statistically significantly attenuated acoustic startle reflex toward smoking‐related versus neutral stimuli (*P* < 0.001, Rosenthal's *r* = 0.39), while no statistically significant differences emerged for other psychophysiological or cognitive‐behavioral measures. Neural measures showed statistically significantly heightened reactivity toward smoking‐related versus neutral stimuli in sensory and motor regions (e.g. precuneus; *P <* 0.001, Rosenthal's *r* = 0.44) but reduced activity in reward‐related regions (e.g. striatum; *P* = 0.021, Cohen's *d* = 0.22). Higher craving was statistically significantly associated with stronger appetitive responses on some measures from all assessment methods (*P*s ≤ 0.041), whereas greater tobacco dependence and smoking behavior were linked to reduced neural reactivity toward smoking‐related stimuli (*P*s ≤ 0.036). No statistically significant associations emerged between measures from different methods (factor loadings ≤ 0.145, *P*s ≥ 0.076). Differences scores between conditions (rel. = −0.351 to 0.837) were generally less reliable than their individual components (rel. = 0.619 to 0.964; excluding one exception)

**Conclusions:**

Appetitive responses toward smoking‐related stimuli may play a limited role in abstinence‐motivated, non‐deprived individuals with chronic tobacco dependence, whereas habitual motor responses could be more crucial.

## INTRODUCTION

Tobacco smoking is a leading risk factor for preventable diseases and pre‐mature deaths [1]. Despite a strong desire to quit, most individuals who smoke experience repeated relapses [2]. The inability to quit, even among highly motivated individuals, is a core feature of tobacco dependence. Prominent theoretical frameworks suggest that smoking is driven by impaired inhibitory control and strong appetitive responses toward smoking‐related cues [3]. Neurobiologically, the incentive‐sensitization theory (IST) [4] hypothesizes that repeated drug use leads to a sensitization of the mesocorticolimbic brain circuitry, increasing the incentive‐motivational value of drug‐related cues (incentive salience). This sensitization may evoke a strong experience of conscious craving, but can also manifest solely through unconscious motivational processes that direct attention to drug‐related cues and elicit appetitive responses, such as strong behavioral approach tendencies [5]. Furthermore, dual‐process models [6, 7] postulate that problematic drug use is driven by an imbalance between weakened controlled and strong impulsive processes. Continued drug use is thought to exacerbate this imbalance so that impulsive processes (e.g. appetitive responses) are increasingly hard to regulate by the more controlled processes (e.g. abstinence motivation).

### Overview of previous research

Various tasks have been developed to assess drug‐induced appetitive responses. Cognitive‐behavioral assessments include the joystick approach‐avoidance task (AAT) [8], which measures behavioral approach tendencies, and (single‐target) Implicit‐Association Tests (ST‐IAT) [9], which capture associative (cognitive) biases (e.g. associations between smoking and approach‐avoidance or valence). Evidence for cognitive‐behavioral approach biases in smoking remains inconsistent [10]. Regarding valence associations, research has frequently shown that individuals who smoke hold negative attitudes toward smoking (e.g. De Houwer *et al*., Wittekind *et al*. and Waters *et al*.) [11, 12, 13]. This seemingly contradictory finding can be explained by the IST, stating that the processes of drug‐liking and drug‐wanting diverge as dependence increases [5]. Appetitive responses have also been assessed psychophysiologically using facial electromyography (EMG). Empirical studies have shown that individuals who smoke exhibit attenuated acoustic startle responses [14] and facial muscle activity (zygomaticus and corrugator muscles) reflecting appetitive responses toward smoking‐related stimuli [15, 16]. Furthermore, meta‐analyses have found heightened neural reactivity in regions involved in reward processing [e.g. anterior cingulate cortex (ACC), striatum] during exposure to smoking‐related stimuli (termed cue‐reactivity) [17, 18]. Regarding impulsive processes, reduced performance in inhibitory control tasks has been linked to smoking behavior [19, 20].

Appetitive responses toward smoking‐related stimuli have attracted substantial research interest, however, several limitations persist. First, many previous studies relied on non‐treatment‐seeking samples and often manipulated satiation (e.g. Wiers *et al*. and Would *et al*.) [21, 22]. Yet, interventions targeting such responses [i.e. cognitive‐bias modification (CBM)] are partly delivered under unrestricted smoking conditions (e.g. Wittekind *et al*. and Smits *et al*.) [12, 23] and considered most effective in individuals motivated to change or quit [24]. Therefore, it is essential to examine whether these processes are engaged in the very group for whom CBM is intended. Second, most studies used a single‐method approach (e.g. cognitive‐behavioral measures). However, to capture the complexity of appetitive responses, a multi‐method approach is recommended, incorporating multiple units of analysis to provide a more comprehensive and valid understanding of the underlying mechanisms [see Research Domain Criteria (RDoC)] [25]. Third, associations between measures of appetitive responses remain largely unexplored, yet it is crucial for testing the IST's assumption of incentive sensitization as their shared mechanism. Findings from the few studies investigating correlations between cognitive‐behavioral (i.e. attentional, approach and associative) biases have been inconsistent [12, 22, 26, 27]. Importantly, no research has yet examined associations using a multi‐method approach, including psychophysiological and neural assessments. Fourth, drawing robust inferences and valid conclusions from statistical findings requires reliable measures, as reliability constrains the observable association between them [28, 29]. To date, the reliability of measures assessing appetitive responses in smoking has only been investigated for cognitive‐behavioral measures (e.g. Wittekind *et al*.) [12], with psychophysiological and neural measures still unexplored.

### The present study

This study examined appetitive responses toward smoking‐related stimuli in a large sample (*n* = 362) of chronically smoking individuals with moderate‐to‐heavy tobacco dependence who were abstinence‐motivated and non‐deprived. Our multi‐method approach included cognitive‐behavioral (AAT, ST‐IATs), psychophysiological (facial EMG) and neural assessments [functional magnetic resonance imaging (fMRI)]. Based on dual‐process models and the IST, we hypothesized that: (1) participants exhibit appetitive responses toward smoking‐related stimuli; (2) higher scores on smoking‐related variables (i.e. cigarettes per day, tobacco dependence severity and current craving) are associated with stronger appetitive responses and lower inhibitory control performance (assessed via Stroop task) [30]; and (3) appetitive responses are interrelated based on incentive sensitization as their shared mechanism. Moreover, the reliability of all measures was evaluated.

## METHODS

This cross‐sectional investigation used baseline data from a pre‐registered intervention study (German Clinical Trials Register: DRKS00019221; 11/11/2019), including the pre‐registration of a cross‐sectional analysis (see Wittekind *et al*. for the study protocol) [31]. The data and analysis code are openly available in OSF: https://osf.io/74ydh/ (dataset) [32]. Appendices can be accessed via the online supporting material.

### Participants

Between November 2019 and March 2023, 362 non‐deprived (i.e. allowed *ad libitum* smoking before assessment) individuals who smoke participated in a pre‐registered intervention study on the efficacy of approach‐bias modification (as specific form of CBM) as an add‐on to smoking cessation treatment. Participants completed the baseline assessment and were included in the present cross‐sectional analysis. Inclusion criteria were: (1) age 18 to 70 years; (2) Fagerström Test for Nicotine Dependence (FTND) [33] score ≥3; 1 (3) exhaled carbon monoxide (CO) ≥ 10 ppm; and (4) smoking ≥10 cigarettes per day within the past 12 months. Exclusion criteria were: (1) current/previous diagnosis of severe psychiatric disorders (bipolar disorder, psychosis); and (2) moderate or severe substance use disorder [SUD; ≥4 Diagnostic and Statistical Manual of Mental Disorders, Fifth Edition (DSM‐IV) criteria met, as assessed with the Mini International Neuropsychiatric Interview (MINI)] [35] other than tobacco within the past 12 months. Full eligibility criteria are provided in Appendix A.1. Between March 2022 and March 2023, a subsample (*n* = 117) participated in an optional fMRI investigation (see Appendix A.1 for fMRI‐specific exclusion criteria). The study was approved by the Ludwig‐Maximilians‐University Munich ethics committee. All individuals provided written informed consent.

### Procedures, questionnaires and experimental paradigms

Socio‐demographic and drug‐related information (e.g. cigarettes per day, time since last cigarette) were collected in a baseline interview, followed by experimental tasks, questionnaires and a psychophysiological assessment (see Appendix A.2 for details and illustration). The fMRI investigation was arranged as a separate appointment. Tobacco dependence 2 was measured using the 12‐item Cigarette Dependence Scale‐12 (CDS‐12) [36] (Cronbach's α = 0.75, 95% CI = 0.72–0.79), aligning with the DSM‐IV and International Statistical Classification of Diseases (ICD)‐10 criteria. Craving was assessed using the Brief Questionnaire of Smoking Urges (QSU‐brief) [37] (Cronbach's α = 0.90, 95% CI = 0.88–0.91) on a 7‐item Likert‐like scale.

Experimental tasks are described in detail in Appendix A.3. Cognitive‐behavioral tasks included: a joystick‐based AAT [8] to assess behavioral approach tendencies by comparing reaction times (RTs) for pushing versus pulling smoking‐related stimuli; two ST‐IATs [9] to evaluate implicit associations between smoking and approach/avoidance or positive/negative valence by comparing RTs in compatible (smoking paired with approach/positive words) versus incompatible trials (smoking paired with avoidance/negative words); and a color Stroop task [30] to assess inhibitory control by comparing RTs in incompatible (ink color–word meaning conflict) versus control trials (color naming). The psychophysiological assessment included facial EMG during the presentation of smoking‐related, neutral, positive and negative pictures [16] to assess muscle activity: corrugator supercilii (over eyebrow; contraction reflects negative valence), zygomaticus major (cheek; contraction reflects positive valence) and orbicularis oculi (under eye) responses during acoustic startle probes (startle attenuation reflects positive valence) [38]. The neural assessment involved an fMRI cue‐reactivity paradigm (presentation of smoking‐related and neutral pictures) [39] to examine brain activity in response to smoking‐related versus neutral stimuli.

### Statistical analysis

#### Data pre‐processing and measure extraction

Data pre‐processings are described in Appendix A.4. The study collected four cognitive‐behavioral (AAT, both ST‐IATs and Stroop task) (Appendix A.4.1) and three psychophysiological measures (EMG: corrugator, zygomaticus and orbicularis oculi) (Appendix A.4.2). To identify brain regions involved in smoking cue‐reactivity [contrast (smoking > neutral)], we combined a hypothesis‐driven region of interest (ROI) and whole‐brain analysis (Appendix A.4.3). ROIs we selected based on Lin *et al*.’s [17] meta‐analysis, including the ACC, left angular gyrus, right thalamus and right striatum. A whole‐brain analysis identified clusters surviving a voxel‐wise threshold of *P* < 0.001 and an extent threshold of family‐wise error‐corrected cluster size (FWEc) = 336 voxels (*P*_FWE_ < 0.05) using SPM12. A one‐sample *t* test on the first‐level contrast images (smoking > neutral), including age and sex as covariates, revealed three regions: right middle cingulate and paracingulate gyri (MCC), right precuneus and right supramarginal gyrus. In total, seven regions relevant to smoking cue‐reactivity were identified.

Condensed, the study included 13 measures assessing responses toward smoking‐related stimuli and one assessing inhibitory control (Stroop task) (see Table 1). Because of the unacceptable/lower split‐half reliability 3 of difference scores (e.g. responses in smoking‐related minus neutral trials) (see Table 1 notes), responses in target conditions were used as outcome measures (AAT: pull_smoking_; ST‐IATs: RTs in compatible trials; Stroop task: incompatible trials; EMG/fMRI: activity during smoking‐related trials) while controlling for the corresponding control conditions (AAT: push_smoking_; ST‐IATs: RTs in incompatible trials; Stroop task: control trials; EMG/fMRI: activity during neutral trials). Pre‐registered difference scores (see Wittekind *et al*.) [31] are reported if they differ from target condition findings. For the zygomaticus activity, the difference score reliability (=0.273) exceeded that of the target condition (=0.113) and was, therefore, retained.

**TABLE 1:** Descriptive statistics, results of difference tests and reliability of measures.

Measures	*n*	Target condition		Control condition		Statistics	Reliability [95% CI]^b^
		M (SD)	Median	M (SD)	Median		
Cognitive‐behavioral							
AAT	357	725.76 (88.66)	719.00	720.54 (82.70)	713.00	*Z* = 1.51, *P* = 0.130	0.964 [0.957–0.970]
Approach ST‐IAT	353	888.54 (173.60)	860.80	884.95 (192.38)	846.36	*Z* = 1.16, *P* = 0.248	0.923 [0.887–0.952]
Valence ST‐IAT	353	866.81 (153.41)	839.71	802.98 (134.24)	778.99	*Z* = 11.30, ***P* < 0.001** ^a^, *r* = 0.60	0.905 [0.883–0.930]
Stroop	352	1388.80 (404.03)	1300.19	1116.01 (284.60)	1063.47	*Z* = 15.30, ***P* < 0.001** ^a^, *r* = 0.82	0.862 [0.840–0.890]
Psychophysiological							
EMGcor	300	0.14 (0.57)	0.06	0.12 (0.53)	0.07	*Z* = 1.85, *P* = 0.064	0.771 [0.588–0.965]
EMGzyg	300	−0.005 (0.47)	−0.003	0.06 (0.58)	0.005	*Z* = ‐1.10, *P* = 0.272	0.273 [−0.001 to 0.598]
EMGstartle	180	32.85 (28.96)	22.13	36.12 (30.90)	26.24	*Z* = –5.16, ***P* < 0.001** ^a^, *r* = 0.39	0.957 [0.940–0.970]
Neural							
ACC	113	−0.03 (0.21)	−0.04	−0.11 (0.22)	−0.14	*t*(112) = 4.70, ***P* < 0.001** ^a^, *d* = 0.44	0.619 [0.507–0.749]
Left angular gyrus	113	0.02 (0.21)	0.002	0.03 (0.24)	0.006	*t*(112) = −0.65, *P* = 0.520	0.635 [0.506–0.768]
Right thalamus	113	0.09 (0.24)	0.09	0.14 (0.24)	0.13	*t*(112) = −3.41, ***P* < 0.001** ^a^, *d* = 0.32	0.694 [0.591–0.810]
Right striatum	113	0.01 (0.15)	−0.02	0.03 (0.16)	0.02	*t*(112) = −2.35, ***P* = 0.021** ^a^, *d* = 0.22	0.659 [0.562–0.756]
MCC	113	0.15 (0.34)	0.16	0.06 (0.36)	0.09	*Z* = 4.76, ***P <* 0.001** ^a^, *r* = 0.45	0.733 [0.627–0.853]
Right precuneus	113	−0.16 (0.32)	−0.14	−0.24 (0.33)	−0.20	*Z* = 4.67, ***P <* 0.001** ^a^, *r* = 0.44	0.817 [0.763–0.863]
Right supramarginal gyrus	113	−0.09 (0.24)	−0.08	−0.18 (0.25)	−0.18	*Z* = 5.70, ***P <* 0.001** ^a^, *r* = 0.54	0.672 [0.531–0.817]

*Note*: Split‐half reliabilities apply to the target condition variable (EMGzyg: difference score). Effect sizes are reported as Cohen's *d* [40] for *t* tests and Rosenthal's *r* [41] for Wilcoxon tests, with 0.20–0.50 indicating small to medium effects. Significant *P* values are indicated in bold.

Abbreviations: AAT, Approach‐Avoidance Task; ACC, left anterior cingulate and paracingulate gyri; CDS‐12, 12‐item Cigarette Dependence Scale; EMG, electromyography; EMGcor, electromyography over the corrugator supercilii muscle; EMGstartle, electromyography over the orbicularis oculi muscle; EMGzyg, Electromyography over the zygomaticus major muscle; fMRI, functional magnetic resonance imaging; FTND, Fagerström Test for Nicotine Dependence; MCC, right middle cingulate and paracingulate gyri; QSU‐brief, Brief Questionnaire of Smoking Urges; ST‐IAT, single‐target Implicit‐Association Test.

^a^ Significant after Benjamini‐Hochberg correction.

^b^ Reliability of difference scores: AAT: 0.837 [0.799–0.871]; approach ST‐IAT: 0.684 [0.636–0.758]; valence ST‐IAT: 0.654 [0.593–0.709]; Stroop task, EMG and fMRI measures: −0.351 to 0.345.

#### Data analysis strategy

Data were analyzed using R, version 4.3.0 [43]. For hypothesis 1, two‐tailed paired non‐parametric Wilcoxon signed‐rank or parametric *t* tests assessed response differences between target and control conditions (zygomaticus difference score: one‐sample Wilcoxon signed‐rank against zero). A power analysis using G*Power [44] determined that *n* = 199 participants are required to detect a small effect (*d* = 0.2) with *α* = 0.05 and 1‐β = 0.80 in a two‐tailed paired *t* test. For hypothesis 2, linear regressions examined associations between smoking‐related variables (cigarettes per day, FTND and QSU‐brief) as predictors and responses in target conditions as outcomes, with age, sex, deprivation, 4 and control condition responses as covariates. For hypothesis 3, confirmatory factor analyses (CFAs) tested whether target condition responses loaded onto a latent factor (incentive sensitization). The latent factor was regressed on age, sex and deprivation, with control condition responses as covariates (see Appendix B for CFA model structure). Measures were *z*‐standardized and reversed (if necessary) to ensure higher scores reflecting stronger appetitive responses. Analyses used the R package *lavaan* [46] with robust maximum likelihood estimation and full information maximum likelihood (FIML) for missing data. Model fit was assessed via robust comparative fit index (CFI) (≥0.95), Tucker‐Lewin index (TLI) (≥0.95), root‐mean‐square error of approximation (RMSEA) (≤0.06) and standardized root‐mean‐square residual (SRMR) (≤0.08) [47] using the *compareFit* function from the *semTools* package [48]. As fMRI data were available only for a subsample (*n* = 113), a second CFA was conducted using only cognitive‐behavioral and psychophysiological measures. Additionally, non‐parametric partial correlations examined associations between target condition measures, controlling for age, sex, deprivation and control condition responses. The Benjamini‐Hochberg correction [49] was applied to control the false discovery rate (FDR) at 5%, adjusting for the 14 tests performed because of the 14 outcome measures.

## RESULTS

### Sample characteristics

Participants were between 20 and 69 years old [mean (M) = 42.33 years, SD = 12.57], with 51.38% female. 5 Most were highly educated [63.97% held a German university entrance level qualification (‘Abitur’); *n* = 4 missing values 6]. On average, participants smoked 18.74 cigarettes daily (SD = 6.53; range: 10–60; *n* = 3 missing values) for 23.64 years (SD = 12.22, range: 1.5–54.5; *n* = 3 missing values) and showed moderate to high tobacco dependence severity (FTND: M = 5.35, SD = 1.60, range: 3–10, scale range: 0–10; CDS‐12: M = 35.81, SD = 5.51, range: 19–48, scale range: 0–48). Craving at assessment start was low on average (QSU‐brief: M = 16.66, SD = 10.88, range: 0–53, scale range: 0–70) with most participants smoking approximately 30 minutes prior (M = 32.63, SD = 54.97, range: 5–630 7).

### Stimulus ratings

Smoking‐related stimuli used in the EMG, fMRI and AAT assessments were rated to elicit moderate craving levels, but significantly higher cravings compared to neutral stimuli (Table 2). Additionally, smoking‐related stimuli from the EMG assessment were rated as significantly more unpleasant and arousing than neutral stimuli.

**TABLE 2:** Stimulus ratings.

Ratings (scale)	Smoking‐related stimuli/blocks	Neutral stimuli/blocks	Statistics
	M (SD)	M (SD)	
Psychophysiological			
Valence [pleasant (1) to unpleasant (9)]	3.87 (1.73)	3.55 (1.51)	*Z* = 3.09, ***P* = 0.002**
Arousal [relaxed (1) to aroused (9)]	3.56 (1.69)	2.84 (1.47)	*Z* = 7.84, ***P* < 0.001**
Craving [not at all (1) to very strongly (9)]	4.60 (2.34)	2.59 (1.57)	*Z* = 13.99, ***P* < 0.001**
Neural^a^			
Craving [‘I want to smoke now’: strongly disagree (0) to totally agree (100)]	54.09 (26.14)	30.62 (20.44)	*Z* = 7.84, ***P* < 0.001**

*Note*: Two‐tailed paired Wilcoxon signed‐rank tests investigated differences in ratings of smoking‐related versus neutral stimuli/blocks. Significant *P* values are indicated in bold.

Abbreviations: AAT, Approach‐Avoidance Task; fMRI, functional magnetic resonance imaging.

^a^ Smoking‐related stimuli from the fMRI smoking cue‐reactivity paradigm were also used in the cognitive‐behavioral AAT.

### Hypothesis 1: Appetitive responses toward smoking‐related stimuli

#### Cognitive‐behavioral measures

Participants did not exhibit significant cognitive (approach ST‐IAT) and behavioral (AAT) approach biases toward smoking‐related stimuli (Table 1). Specifically, push and pull RTs for smoking‐related stimuli in the AAT were not significantly different, nor did participants associate smoking more strongly with approach than avoidance in the ST‐IAT. However, participants responded faster when smoking and negative attributes shared a response key compared to smoking and positive attributes (negative valence ST‐IAT bias). As expected, participants were significantly slower in incompatible trials (ink color–word meaning conflict) compared to control trials (color naming).

#### Psychophysiological measures

Participants exhibited a significantly attenuated acoustic startle response (EMGstartle) during smoking‐related compared to neutral trials (Table 1). However, corrugator (EMGcor) and zygomaticus (EMGzyg) muscle responses did not differ significantly, indicating similar activity levels in smoking‐related versus neutral trials.

#### Neural measures

Participants showed significantly higher brain activity during exposure to smoking‐related compared to neutral stimuli in the ACC, MCC, right precuneus and right supramarginal gyrus (Table 1). Conversely, activity in the right thalamus and right striatum was significantly lower for smoking‐related versus neutral stimuli. No significant difference was observed for the left angular gyrus.

### Hypothesis 2: Associations between appetitive responses and inhibitory control with smoking‐related variables 8

#### Cognitive‐behavioral measures

Linear regression results (Table 3) showed no significant associations between daily cigarette consumption, tobacco dependence severity or craving and cognitive‐behavioral measures of appetitive responses toward smoking‐related stimuli (AAT, both ST‐IATs) or inhibitory control performance (Stroop). This suggests that heavier smoking, greater dependence and stronger craving were not linked to faster pull RTs for smoking‐related stimuli (AAT), stronger smoking‐approach or smoking‐positive associations (ST‐IATs) or slower responses in incompatible Stroop trials. However, stronger craving was significantly associated with stronger smoking‐approach associations when using the approach ST‐IAT difference score as the outcome (see Table 3 notes).

**TABLE 3:** Linear regression results: Associations between appetitive responses, inhibitory control and smoking‐related variables.

Measures	Cigarettes per day			Tobacco dependence (CDS‐12)			Craving^b^		
	β	95% CI	*P*	β	95% CI	*P*	β	95% CI	*P*
Cognitive‐behavioral									
AAT	−0.396	−1.335 to 0.543	0.408	−0.343	−1.470 to 0.785	0.550	0.191	−0.356 to 0.739	0.493
Approach ST‐IAT	−0.827	−2.805 to 1.151	0.412	−0.311	−2.679 to 2.058	0.797	−0.897	−2.056 to 0.261	0.128^c^
Valence ST‐IAT	−0.671	−2.373 to 1.031	0.439	−0.204	−2.244 to 1.836	0.844	−0.742	−1.740 to 0.256	0.145
Stroop	3.360	−0.566 to 7.285	0.093	2.249	−2.448 to 6.946	0.347	1.081	−1.185 to 3.346	0.349
Psychophysiological									
EMGcor	−0.006	−0.014 to 0.003	0.177	−0.004	−0.014 to 0.006	0.480	−0.005	−0.010 to 0.0003	**0.038**
EMGzyg	0.008	−0.005 to 0.022	0.209	0.010	−0.005 to 0.026	0.198	0.010	0.002–0.017	**0.015**
EMGstartle	−0.021	−0.245 to 0.204	0.857	0.030	−0.203 to 0.263	0.798	0.059	−0.051 to 0.168	0.293
Neural									
ACC	−0.003	−0.008 to 0.001	0.160	0.001	−0.004 to 0.006	0.757	0.001	0.0002–0.002	0.113
Left angular gyrus	−0.004	−0.009 to 0.001	0.085	−0.003	−0.007 to 0.002	0.301	0.001	0.0004–0.002	0.178
Right thalamus	−0.005	−0.009 to −0.0003	**0**.**036**	−0.003	−0.008 to 0.002	0.203	0.001	0.0001–0.002	0.070
Right striatum	−0.002	−0.005 to 0.001	0.131	−0.001	−0.004 to 0.002	0.445	0.001	0.00003–0.001	**0**.**041**
MCC	−0.009	−0.015 to −0.003	**0.005** ^**a**^	−0.009	−0.016 to −0.002	**0.009**	0.001	−0.001 to 0.002	0.531
Right precuneus	−0.007	−0.012 to −0.002	**0.008** ^**a**^	−0.008	−0.014 to −0.002	**0.006**	0.0003	−0.002 to 0.001	0.632
Right supramarginal gyrus	−0.006	−0.011 to −0.002	**0.003** ^**a**^	−0.005	−0.010 to −0.0005	**0.031**	0.00003	−0.001 to 0.001	0.961

*Note*: Linear regressions were performed with age, sex and deprivation (not applicable for neural measures and not included in models where craving was the main predictor) as covariates. Predictors, except for sex, were grand‐mean centered. Significant *P* values are indicated in bold.

Abbreviations: AAT, Approach‐Avoidance Task; ACC, left anterior cingulate and paracingulate gyri; CDS‐12, 12‐item Cigarette Dependence Scale; EMGcor, electromyography over the corrugator supercilii muscle; EMGstartle, electromyography over the orbicularis oculi muscle; EMGzyg, electromyography over the zygomaticus major muscle; MCC, right middle cingulate and paracingulate gyri; QSU‐brief, Brief Questionnaire of Smoking Urges; ST‐IAT, single‐target Implicit‐Association Test.

^a^ Significant after Benjamini‐Hochberg correction.

^b^ For cognitive‐behavioral and psychophysiological measures, the QSU‐brief score was used as predictor. For neural measures, the mean craving rating after smoking‐related blocks was used, as the QSU‐brief was not administered during the fMRI session.

^c^ Using the approach ST‐IAT difference score, higher craving was significantly associated with a cognitive approach bias toward smoking‐related stimuli (β = 0.004, 95% CI = 0.0004–0.008, *P* = 0.030).

#### Psychophysiological measures

Higher craving was significantly associated with reduced corrugator (EMGcor) and increased zygomaticus activity (EMGzyg) in response to smoking‐related stimuli (EMGzyg: relative to neural stimuli), although both effects were non‐significant after FDR‐correction (Table 3). This suggests that participants with stronger craving tended to exhibit lower corrugator and higher zygomaticus activity toward smoking‐related stimuli. Craving was not significantly associated with the acoustic startle response (EMGstartle), indicating no relationship with the startle reflex during smoking‐related trials. Additionally, no significant associations were found between facial muscle activity and daily cigarettes or dependence severity, suggesting that smoking heaviness and dependence were unrelated to zygomaticus and corrugator activity, and the acoustic startle reflex during smoking‐related trials.

#### Neural measures

Higher daily cigarette consumption was significantly associated with reduced brain activity in the right thalamus (non‐significant after FDR‐correction), MCC, right precuneus and right supramarginal gyrus in response to smoking‐related stimuli (Table 3). This suggests that participants with higher consumption exhibited lower activity in these regions when exposed to smoking‐related stimuli compared to those with lower consumption. Similarly, greater dependence severity was significantly associated with reduced activity in the MCC, right precuneus and right supramarginal gyrus (all non‐significant after FDR‐correction), indicating lower activity in these regions among participants with greater dependence. Last, higher craving was significantly associated with increased activity in the right striatum (non‐significant after FDR‐correction), indicating heightened striatal activity among participants with stronger craving. No significant associations were observed for other brain regions.

### Hypothesis 3: Interrelations between measures of appetitive responses and inhibitory control

The CFAs showed that cognitive‐behavioral and neural measures loaded significantly onto separate latent factors (Table 4) (AAT and ST‐IAT difference scores did not load onto a shared latent factor). Importantly, no significant cross‐loadings between measures from different assessment methods were observed. Moreover, model fit indices indicated misspecification, suggesting no shared latent factor across measures. Partial correlations supported the CFA results, showing no significant associations between measures from different assessment methods (with one exception; see Appendix D).

**TABLE 4:** Results of the confirmatory factor analyses.

Measures	CFA across cognitive‐behavioral, psychophysiological and neural measures^b^		CFA across cognitive‐behavioral and psychophysiological measures^c^ ^,^ ^d^	
	Loading (SE)	*P*	Loading (SE)	*P*
Cognitive‐behavioral				
AAT	0.145 (0.082)	0.076	0.173 (0.060)	**0**.**004** ^a^
Approach ST‐IAT	0.145 (0.113)	0.198	0.336 (0.085)	**<0.001** ^a^
Valence ST‐IAT	0.063 (0.080)	0.429	0.277 (0.067)	**<0.001** ^a^
Psychophysiological				
EMGcor	−0.009 (0.139)	0.949	−0.008 (0.113)	0.947
EMGzyg	−0.069 (0.156)	0.660	0.087 (0.121)	0.471
EMGstartle	−0.043 (0.051)	0.406	0.035 (0.039)	0.377
Neural				
ACC	−0.522 (0.110)	**<0.001** ^a^	–	–
Left angular gyrus	−0.451 (0.122)	**<0.001** ^a^	–	–
Right thalamus	−0.524 (0.071)	**<0.001** ^a^	–	–
Right striatum	−0.497 (0.062)	**<0.001** ^a^	–	–
MCC	−0.350 (0.087)	**<0.001** ^a^	–	–
Right precuneus	−0.262 (0.105)	**0**.**013** ^a^	–	–
Right supramarginal gyrus	−0.452 (0.082)	**<0.001** ^a^	–	–

*Note*: Model fit indices indicate a poor fit for both models ([47]; recommended cut‐off criteria: CFI ≥ 0.95, TLI ≥ 0.95, RMSEA ≤ 0.06 and SRMR ≤ 0.08). Significant *P* values are indicated in bold.

Abbreviations: AAT, Approach‐Avoidance Task; ACC, left anterior cingulate and paracingulate gyri; CFA, confirmatory factor analysis; CFI, comparative fit index; EMGcor, electromyography over the corrugator supercilii muscle; EMGstartle, electromyography over the orbicularis oculi muscle; EMGzyg, electromyography over the zygomaticus major muscle; MCC, right middle cingulate and paracingulate gyri; RMSEA, root‐mean‐square error of approximation; SE, standard error; SRMR, standardized root‐mean‐square residual; ST‐IAT, single‐target Implicit‐Association Test; TLI, Tucker‐Lewin index.

^a^ Significant after Benjamini‐Hochberg correction.

^b^ χ^2^(347) = 1561.79, *P <* 0.001; CFI = 0.63; TLI = 0.60; RMSEA = 0.17; SRMR = 0.26.

^c^ χ^2^(74) = 527.19, *P <* 0.001; CFI = 0.80; TLI = 0.76; RMSEA = 0.14; SRMR = 0.18.

^d^ No significant loadings were found when using the AAT and ST‐IAT difference scores as outcome measures (all *P* ≥ 0.084).

## DISCUSSION

This study was the first to investigate appetitive responses toward smoking‐related stimuli in a large sample of abstinence‐motivated, non‐deprived individuals with chronic, moderate‐to‐heavy tobacco dependence using a multi‐method experimental approach. All measures showed good to excellent reliability, except for the measure assessing zygomaticus muscle activity, for which results should be interpreted with caution [28]. Overall, findings indicate that, under the applied experimental paradigms, appetitive responses were not strongly engaged in abstinence‐motivated, non‐deprived individuals with chronic tobacco dependence, providing no support for the core assumptions of the IST.

### Summary of findings

#### Hypothesis 1: Appetitive responses toward smoking‐related stimuli

A central tenet of the IST posits that individuals who smoke exhibit appetitive responses toward smoking‐related stimuli, however, this is largely unsupported by our findings in the present sample. Specifically, appetitive responses were not observed with cognitive‐behavioral or psychophysiological measures, except for an attenuated acoustic startle reflex when exposed to smoking‐related compared to neutral stimuli. This exceptional finding suggests that smoking cues hold positive significance for individuals who smoke—at least when assessed via the acoustic startle reflex. Regarding valence associations, our findings revealed strong negative attitudes toward smoking, consistent with prior research [11, 12, 13]. This aligns with the IST assumption that drug‐liking and ‐wanting can be dissociated in chronic substance dependence.

Consistent with meta‐analytical findings [17, 18], participants showed heightened brain reactivity in the ACC, MCC, right precuneus and right supramarginal gyrus in response to smoking‐related compared to neutral stimuli. The cingulate gyrus (encompassing the ACC and MCC), a key cortical region in smoking cue‐reactivity [17], is involved in emotional processing and motor coordination and control [50, 51]. However, subcortical mesolimbic structures, such as the thalamus and striatum, showed reduced activity in response to smoking‐related compared to neutral stimuli. This is significant, as the IST posits that incentive salience processes are mediated by heightened activity in subcortical mesolimbic structures [5]. Therefore, smoking‐related stimuli may not be processed as rewarding [52, 53]. Instead, the observed increased reactivity in sensory and motor brain regions, such as the right precuneus and right supramarginal gyrus, rather reflects attentional bias and automatized, habitual motor responses toward smoking‐related stimuli [54].

#### Hypothesis 2: Associations between appetitive responses and inhibitory control with smoking‐related variables

The findings challenge the assumption of the IST that heavier smoking and greater tobacco dependence are associated with stronger appetitive responses toward smoking‐related stimuli. Instead, neural measures revealed the opposite, heavier smoking and greater dependence were associated with reduced activity in sensory and motor regions (MCC, precuneus and supramarginal gyrus). This may indicate that, under certain conditions (e.g. non‐deprived state), individuals who smoke more heavily and are more dependent consume cigarettes more automatically, relying less on external cues. In contrast, moderately dependent individuals might be more distracted and exhibit stronger motor responses toward smoking cues. The negative association between thalamic activity and heavier smoking (non‐significant after FDR‐correction) aligns with prior research linking greater dependence severity to reduced activation in subcortical mesolimbic structures (e.g. striatum, amygdala and putamen) [17, 55]. Therefore, with heavier smoking, mesolimbic structures may exhibit hypoactivity toward smoking cues, contrasting with the premise of the IST that ongoing drug use leads to a hyperactivation of the mesolimbic system. Under certain conditions, heavy smoking behavior may, therefore, involve reduced reliance on incentive‐driven cue processing.

According to the IST, higher experienced (conscious) craving may, but does not necessarily, correspond to stronger unconscious appetitive responses. Our findings partially support such associations (all effects non‐significant after FDR‐correction): higher craving was related to stronger cognitive approach biases, 9 increased zygomaticus activity (positive valence), reduced corrugator activity (reduced negative valence) and increased striatal activity (reward processing) in response to smoking‐related stimuli. These results provide tentative support for a link between conscious craving and unconscious incentive salience processes, at least for some measures.

Regarding impulsive processes, dual‐process models propose an association between lower inhibitory control and heavier smoking, tobacco dependence and craving. However, Stroop task performance was not significantly associated with smoking‐related variables. Nevertheless, as the Stroop task targets interference inhibition [56], future research should examine tasks addressing other facets of inhibitory control, such as action restraint (Go/No‐Go Task) or action cancellation (Stop‐Signal Task) [57].

#### Hypothesis 3: Interrelations between measures of appetitive responses and inhibitory control

According to the IST, appetitive responses toward drug‐related stimuli are driven by increased incentive sensitization as a shared mechanism. However, findings from CFAs and partial correlations do not support this. Measures from different assessment methods neither loaded significantly onto a shared latent factor (CFAs) nor correlated with each other (partial correlations with one exception, see Appendix D). Instead, neural and cognitive‐behavioral 10 measures clustered on separate latent factors or intercorrelated within the same assessment method. This may reflect shared response tendencies within methods (e.g. approach biases driving both cognitive and behavioral tendencies, or neural co‐activation across regions) or shared measurement errors. Regardless, the multi‐method approach provides limited evidence for a shared mechanism. However, this conclusion is constrained by the general lack of incentive salience effects in this sample. Moreover, the absence of cross‐method associations may also reflect limitations in the sensitivity of current measures to detect incentive salience processes (see Tibboel *et al*., for a critical discussion of the construct validity of cognitive‐behavioral tasks) [58].

### Reliability of appetitive response measures

To our knowledge, this is the first study investigating the split‐half reliability of psychophysiological and neural measures in smoking cue‐reactivity paradigms. Consistent with psychometric research (e.g. Hedge *et al*.) [28], difference scores showed low reliability, whereas their individual components exhibited much better reliability, aligning with findings on test–retest reliability in fMRI alcohol cue‐reactivity paradigms [59]. Low reliability is critical, because it can generate spurious findings or obscure true effects [29, 60]. Our results highlight the importance of assessing and reporting the reliability of measures in substance use disorder (SUD) research to validate statistical findings and improve measure reliability (e.g. through optimizing pre‐processing procedures, see Kahveci *et al*.) [61].

### Implications

#### Theoretical implications

Our results raise the question of whether alternative SUD models might better explain the present findings. Some theoretical accounts posit reduced reward‐related neural processing in chronic drug use, either because of general deficits in recruiting brain reward pathways (reward deficiency syndrome) [62] or a progressive decline in drug‐induced reward (opponent process theory) [63]. However, these models do not readily explain the heightened motor‐region reactivity observed in our data. This gap is addressed by habit models [64, 65], which propose that incentive salience processes drive smoking in the early stages, but diminish as smoking becomes habitual, with automatized motor responses toward smoking cues taking over. This may explain the lack of evidence for appetitive responses in our sample, the negative correlation between dependence severity and subcortical mesolimbic activity (i.e. thalamic regions), and heightened activity in motor regions. Notably, the negative association between dependence severity and activity in motor regions suggests reduced reliance on external cues for smoking behavior at high dependence levels. Future research should test this hypothesis and other theoretical assumptions (e.g. habit models) in smoking.

Beyond alternative theoretical accounts, it is also possible that the assumptions of the IST are not universally applicable to all individuals who smoke and under all conditions [5]. For example, reduced psychophysiological and neural cue‐reactivity has been observed in those motivated to quit smoking [66, 67, 68, 69]. As our sample came from a cessation intervention study, abstinence motivation is likely, although unmeasured. Another possibility is that appetitive responses are less likely to manifest when individuals are nicotine‐satiated and experience low conscious craving. Nevertheless, examining individuals after *ad libitum* smoking remains a common approach (e.g. Lin *et al*., Mogg *et al*. and Rougier *et al*.) [17, 27, 70], as the IST explicitly posits that appetitive responses should emerge independently of conscious desire. Taken together, further research should explore when appetitive responses toward smoking‐related stimuli occur (e.g. in individuals not motivated to quit) and which factors influence their strength (e.g. craving levels). For this purpose, ecologically momentary assessment studies could capture real‐time fluctuations in craving, abstinence motivation and appetitive responses in naturalistic settings.

#### Clinical implications

Findings from abstinence‐motivated individuals eligible for approach‐bias modification yield important clinical implications. Our findings challenge the rationale of such training, which aims to reduce appetitive responses toward drug‐related stimuli [71]. Indeed, meta‐analyses on the efficacy of CBM, including approach‐bias modification, report inconsistent and modest effects in smoking [72]. However, our results suggest that it may be worth exploring its efficacy in individuals experiencing heightened smoking desire. Furthermore, our neural findings point to impulsive, automatized motor processes rather than appetitive (incentive‐driven) responses toward smoking‐related stimuli. This highlights the potential of interventions targeting inhibitory deficits.

### Limitations

Our results should be interpreted against important limitations. First, participants smoked *ad libitum* prior the assessments, with many smoking immediately beforehand (see sample characteristics). This meant they were mostly non‐deprived (satiated) and experienced little conscious desire to smoke when exposed to smoking‐related stimuli, potentially attenuating appetitive responses. However, according to the IST, unconscious appetitive responses can occur even without the conscious experience of craving, such as under satiated conditions [4, 5]. Second, the smoking‐related stimuli used in the EMG, fMRI and AAT assessments were rated to elicit moderate craving, which might be associated with attenuated appetitive responses. However, they were selected from previous studies showing appetitive responses [16, 73, 74] (see Appendix A.3 for picture selection) and were rated as inducing stronger craving than neutral stimuli. Moreover, appetitive responses were found even when craving ratings were moderate [75, 76]. In conjunction with the first limitation, to better determine whether nicotine‐satiation and moderate craving ratings contributed to some null findings, future research should clarify under which conditions self‐reported craving aligns with, or dissociates from, unconscious appetitive responses—for example, by manipulating deprivation status. Third, as shown in the study procedure (Figure A.2.1), craving (QSU‐brief) was assessed only at the beginning, whereas the AAT and both IATs were completed approximately 1 to 1.5 hours later. As craving may have changed differently across individuals during the session, this could have biased the association with cognitive‐behavioral measures. Fourth, the study lacked controls without a smoking history and a control condition with rewarding non‐smoking stimuli (e.g. money). Therefore, it remains unresolved whether individuals who smoke differ in their responses from controls and whether findings are smoking‐specific or reflect more general reward‐related processes. Last, sampling bias cannot be excluded, as participants were recruited for a smoking cessation study. Additionally, data on race or ethnicity were not collected, and the sample's demographics (more females, higher education levels) differ from the broader German smoking population [77], limiting generalizability. Taken together, it is important to acknowledge that our findings may primarily apply to a subgroup of chronically smoking, nicotine‐satiated individuals motivated to quit and characterized by specific demographics (e.g. higher education).

## CONCLUSION

This study is the first to investigate appetitive responses in smoking using a multi‐method approach. Overall, the results provide limited evidence to suggest that appetitive responses play a significant role in abstinence‐motivated, non‐deprived, moderate‐to‐heavy tobacco‐dependent adults with chronic smoking behavior; rather, they suggest the importance of attentional and motor responses. Future research should further examine the assumptions of habit models and explore factors associated with the strength of appetitive responses (e.g. abstinence motivation, craving levels). Last, training interventions should focus on disrupting smoking cue‐induced strong automatized (habitual) motor responses.

## AUTHOR CONTRIBUTIONS

**Franziska Motka:** Data curation (equal); formal analysis (lead); investigation (equal); methodology (equal); project administration (supporting); validation (equal); visualization (equal); writing—original draft (lead); writing—review and editing (equal). **Haoye Tan:** Formal analysis (supporting); writing—review and editing (supporting). **Seth M. Levine:** Formal analysis (supporting); writing—review and editing (supporting). **Sabine Vollstädt‐Klein:** Resources (equal); software (equal); writing—review and editing (equal). **Sarah K. Danböck:** Supervision (supporting); writing—review and editing (equal). **Katja Bertsch:** Conceptualization (equal); funding acquisition (equal); resources (equal); supervision (supporting); writing—review and editing (equal). **Markus H. Winkler:** Methodology (equal); resources (supporting); software (supporting); writing—review and editing (equal). **Charlotte E. Wittekind:** Conceptualization (lead); data curation (equal); funding acquisition (lead); investigation (equal); methodology (equal); project administration (lead); resources (equal); software (equal); supervision (lead); validation (equal); writing—review and editing (equal).

## DECLARATION OF INTERESTS

None.

## Supporting information

Appendix A: Methods
Appendix A.1 Participants
Appendix A.2 Procedures
Figure A.2.1 Study procedure: Overview
Appendix A.3 Experimental tasks and paradigms
Appendix A.3.1 Cognitive‐behavioral tasks
Appendix A.3.2 Psychophysiological assessment
Appendix A.3.3 Functional MRI assessment
Figure A.3.3.1 fMRI smoking cue‐reactivity task design
Appendix A.4 Data preprocessing and measure extraction
Appendix A.4.1 Cognitive‐behavioral tasks
Appendix A.4.2 Psychophysiological assessment
Appendix A.4.3 Functional MRI assessment
Appendix B: Statistical analysis
Figure B.1 Model structure of the confirmatory factor analysis across all measures
Appendix C: Corona pandemic specifics
Appendix D: Results of partial correlations
Table D.1 Partial correlations between measures

## Data Availability

The data and analysis code are openly available in OSF: https://osf.io/74ydh/.

## Footnotes

^1^
A FTND score of 3 or higher indicates at least moderate dependence, see AWMF [34].

^2^
The FTND was used for screening because of its efficiency but demonstrated unacceptable reliability in this study (Cronbach's α = 0.43, 95% CI = 0.34–0.50). Consequently, the CDS‐12 score was used as a measure for dependence severity in all analyses.

^3^
Estimated using the R package *splithalfr* [42] with 5000 random splits and corrected using the Spearman‐Brown formula.

^4^
Deprivation (i.e. time since last cigarette in minutes) was not included as control variable in analyses with fMRI measures because it was not assessed during the fMRI session. Additionally, deprivation was excluded from analyses involving the QSU‐brief score, as craving has been shown to be associated with longer deprivation from smoking [45].

^5^
Data on race or ethnicity were not collected (see Limitations).

^6^
Missing data on some variables resulted from test instructor errors during the socio‐demographic interview.

^7^
For participants who could not specify the time of their last cigarette on the day before the baseline assessment (*n* = 3), it was defaulted to 12:00 pm.

^8^
Our study was conducted before, during and after the coronavirus disease 2019 pandemic. As social restrictions and related factors may have influenced the findings, we examined whether pandemic‐related conditions affected the results. Overall, this was not the case: results remained unchanged when controlling for these conditions (for details, see Appendix C).

^9^
Please note that this association emerged only when the approach ST‐IAT difference score was used as the outcome measure.

^10^
Please note that cognitive‐behavioral measures did not significantly load onto a latent factor when difference scores were used as the outcome measure.
