# Supplementary material for: Appetitive responses toward smoking‐related stimuli in abstinence‐motivated, non‐deprived individuals with chronic tobacco dependence: A multi‐methodological investigation
Source: Addiction. 2025 Dec 14;121(5):1140–52. doi: 10.1111/add.70283 (PMC13088940; doi:10.1111/add.70283)
Supplement: Supplementary file 1 — Appendix A: Methods Appendix A.1 Participants Appendix A.2 Procedures Figure A.2.1 Study procedure: Overview Appendix A.3 Experimental tasks and paradigms Appendix A.3.1 Cognitive‐behavioral tasks Appendix A.3.2 Psychophysiological assessment Appendix A.3.3 Functional MRI assessment Figure A.3.3.1 fMRI smoking cue‐reactivity task design Appendix A.4 Data preprocessing and measure extraction Appendix A.4.1 Cognitive‐behavioral tasks Appendix A.4.2 Psychophysiological assessment Appendix A.4.3 Functional MRI assessment Appendix B: Statistical analysis Figure B.1 Model structure of the confirmatory factor analysis across all measures Appendix C: Corona pandemic specifics Appendix D: Results of partial correlations Table D.1 Partial correlations between measures [file ADD-121-1140-s001.pdf]

Appetitive responses toward smoking-related stimuli in abstinence-motivated, non-deprived individuals with chronic tobacco dependence: A multi-methodological investigation

Supplementary Material

Table of contents

Appendix A: Methods 2

Appendix A.1 Participants 2

Appendix A.2 Procedures 3

Appendix A.3 Experimental tasks and paradigms 5

Appendix A.3.1 Cognitive-behavioral tasks 5

Appendix A.3.2 Psychophysiological assessment 7

Appendix A.3.3 Functional MRI assessment 8

Appendix A.4 Data preprocessing and measure extraction 10

Appendix A.4.1 Cognitive-behavioral tasks 10

Appendix A.4.2 Psychophysiological assessment 11

Appendix A.4.3 Functional MRI assessment 12

Appendix B: Statistical analysis 14

Appendix C: Corona pandemic specifics 15

Appendix D: Results of partial correlations 16

References 19

Appendix A: Methods

Given the extensive methodological details of our multi-method approach, additional information not critical to the main manuscript is provided here.

Appendix A.1 Participants

Participants were recruited through various means, including social media advertisements, online marketing, flyers distributed at medical practices and local universities, as well as through newsletters. In addition to the inclusion criteria outlined in the main manuscript, further criteria included: (5) willingness to abstain from nicotine replacement therapy, e-cigarettes, and any other smoking cessation intervention during study participation, and (6) motivation to participate in the intervention study. Additional exclusion criteria were as follows: (3) current/previous diagnosis of major neurological disorders (e.g., multiple sclerosis, Parkinson's disease), (4) use of nicotine replacement therapy or pharmacological smoking cessation treatments within three months prior to study participation, (5) acute suicidality, (6) current pregnancy or nursing period, and (7) insufficient German language skills. Additional exclusion criteria for the fMRI investigation included: (1) standard MRI contraindications (e.g., pacemaker), (2) current use of psychotropic medication, (3) uncorrectable visual impairments, and (4) a history of head trauma.

The COVID-19 restrictions led to the cancellation of one smoke-free course, which was the treatment-as-usual intervention in the main clinical trial. As a result, $n = 8$ participants underwent the pre-assessment but were not randomized for intervention in the clinical trial. Therefore, the present study included a total of $N = 362$ participants, with $N = 354$ included in the main clinical trial (see German Clinical Trials Register: DRKS00019221; 11/11/2019).

Appendix A.2 Procedures

All participants underwent an initial telephone screening, which included an explanation of the study procedure, verification of inclusion and exclusion criteria, and scheduling of the baseline assessment. The fulfillment of inclusion criteria was verified at the beginning of the baseline assessment through the FTND and CO values. Participants opting for the fMRI investigation attended a separate appointment, which was scheduled first. In this case, inclusion criteria were verified prior to the start of the fMRI session (see Figure A.2.1).

Figure A.2.1

Study procedure: Overview

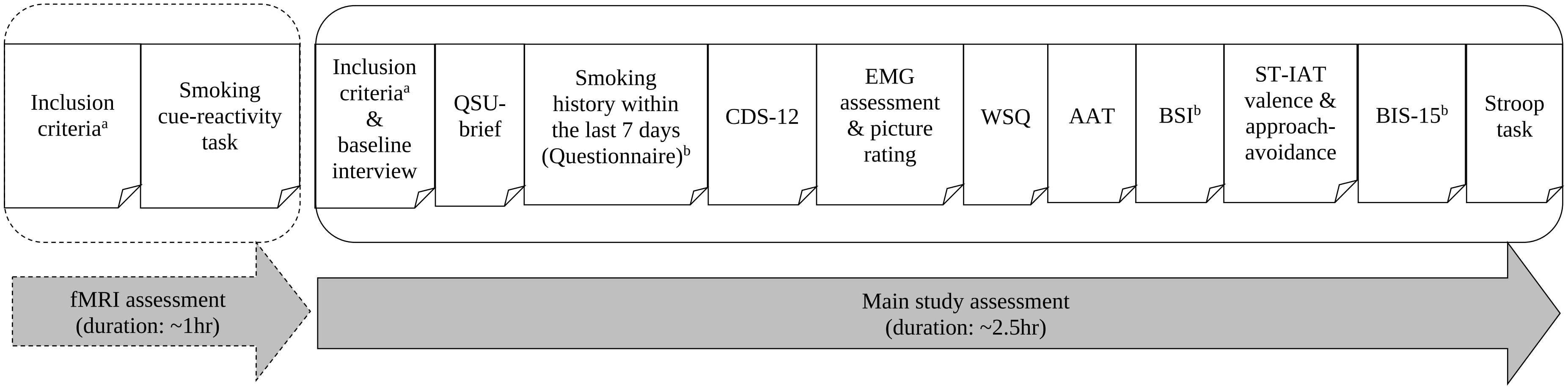

Note. The fMRI investigation was optional (indicated by dashed lines) in addition to study participation and took place between March 2022 and March 2023. fMRI = functional Magnet Resonance Imaging; QSU-brief = Brief Questionnaire of Smoking Urges; CDS-12 = Cigarette Dependence Scale-12; EMG = Electromyography; WSQ = Web Screening Questionnaire; AAT = Approach-Avoidance Task; BSI = Brief Symptom Inventory; ST-IAT = Single-Target Implicit-Association Test; BIS-15 = Barratt Impulsiveness Scale-15.

[a] Carbon monoxide value in exhaled air (CO value) ≥ 10 and Fagerström Test for Nicotine Dependence (FTND) ≥ 3.

[b] Not relevant for the current study.

Appendix A.3 Experimental tasks and paradigms

Appendix A.3.1 Cognitive-behavioral tasks

All cognitive-behavioral tasks were administered on a computer using Inquisit ® Version 4 (www.millisecond.com).

Approach-Avoidance Task (AAT). A joystick-based AAT [1] was employed to measure behavioral approach tendencies. The task comprised a total of 40 smoking-related and 40 positive stimuli. Smoking-related stimuli, featuring pictures of (burning) cigarettes and cigarette packages, were sourced from previous studies [2,3], as well as from online platforms and picture agencies. The stimuli were presented in a pseudo-randomized order (no more than three pictures of the same category were presented consecutively) in two separate blocks (trials per block: n = 80).

A content-relevant feature task instruction was used, directing participants to push or pull the joystick depending on the content of the picture (i.e., push smoking-related pictures and pull positive pictures or vice versa). The order of instruction (push smoking-related pictures first [incongruent task instruction] or pull smoking-related pictures first [congruent task instruction]) was counterbalanced across participants. The instruction switched after the first block. Response direction was linked to a "zoom"-function, such that pushing the joystick decreased the picture size, while pulling the joystick increased it. At the beginning of each test trial, a picture was presented centrally on the screen. Depending on the picture content, participants had to push or pull the joystick. Pictures only disappeared after the joystick had been fully extended in the correct direction; if moved in the wrong direction, the picture remained on the screen until the correct movement was executed. To initiate the next trial, participants were required to press the "fire" button while the joystick was in the central position. Before each block, participants completed six practice trials, resulting in a total of 172 trials.

Single-Target Implicit-Association Test (ST-IAT). Two ST-IATs [4] were employed to assess implicit associations between smoking (target category) and approach/avoidance, as well as positive/negative valence (attribute categories). The attribute categories included six German words related to approach and avoidance as well as six positively and negatively valenced words. The target category contained six German words related to smoking. All stimulus words are provided in the supplemental material of Wittekind et al. [5].

Both IATs consisted of five blocks. The stimulus word for each trial was presented centrally on a black screen in white ink. The labels of the attribute categories (German words for "approach" or "positive" versus "avoid" or "negative", written in green ink) and the target category (the German word for "smoking", written in white ink) were displayed in the upper corners of the screen. During the first block (attribute practice block: n = 12), attribute words were presented and participants were required to classify them using one of two response keys (i.e., "E" or "I"). In the second (practice combined block: n = 24) and third block (test combined block: n = 48), target and attribute words were presented and had to be classified using the same response keys (i.e., "E" for smoking-related and approach/positive words, "I" for avoidance/negative words; compatible condition). In the fourth (practice reversed combined block: n = 24) and fifth block (test reversed combined block: n = 48), the target category (i.e., "smoking") switched positions (i.e., "E" for approach/positive words, "I" for smoking-related and avoidance/negative words; incompatible condition). The order of conditions (congruent blocks first versus incongruent blocks first) was counterbalanced across participants, and the presentation of stimuli was randomized within each block. If the participant mistakenly pressed the wrong key, a red X appeared for 200 ms, and the correct key had to be pressed to proceed to the next trial.

Stroop Task. The color Stroop task [6] was employed to assess participants' inhibitory control performance. Four color words as well as rectangles were presented, printed

in red, green, blue, or black. The task consisted of three conditions (a: congruent trials [word = print color]; b: incongruent trials [word ≠ print color]; c: control trials [colored rectangles]) with a total of 72 trials (4 colors × 3 conditions × 6 repetitions). The trials were arranged in a fully randomized order with an inter-trial interval of 200 ms. During each trial, stimuli were presented at the center of the screen with key assignments displayed in the upper part of the screen (d = red; f = green; j = blue; k = black). Participants were instructed to indicate the print color of either the word or rectangle as quickly and accurately as possible by pressing one of four designated keys on the computer keyboard. After incorrect responses, a red X appeared for 400 ms and the next trial began.

Appendix A.3.2 Psychophysiological assessment

The psychophysiological assessment was implemented through the computer software Presentation (Neurobehavioral Systems). Electromyography (EMG) was used to measure the activity of three facial muscles during a passive picture-viewing task [7]: (1) *Musculus orbicularis oculi* (to assess the acoustic startle reflex), (2) *Musculus corrugator supercilii*, and (3) *Musculus zygomaticus major*. The stimuli were colored pictures from four distinct categories (neutral/positive/negative/smoking-related, n = 12 pictures per category). The smoking-related pictures were selected from previous studies [7,8] and an unpublished picture set by Mucha and Pauli. The pictures depicted events related to the initial stages of smoking, as previous research has indicated that these stimuli elicit stronger physiological responses associated with reward [8,9]. Neutral, positive, and negative stimuli were taken from the International Affective Picture System (IAPS, [10]). Positive and negative stimuli were matched to smoking-related stimuli according to the arousal and absolute valence ratings derived from the IAPS database.

The task was divided into three blocks with four pictures of each category per block (i.e., 16 pictures per block). In each trial, a picture was centrally presented on a black screen for an average of 7.5 s (range: 7.0 to 8.0 s), followed by a black screen for 16.5 to 25.5 s (M =

21.0 s, inter-trial interval). During each block, an acoustic startle response was elicited through headphones, 2.5, 4.0, or 5.5 s after picture onset during three trials of each picture category (tone duration: 50 ms; volume: 105 dB; see Wittekind et al. [5] for further technical details). The order of picture category (neutral/positive/negative/smoking-related), the presentation of the startle stimulus (present/not-present), and its presentation time (2.5 s/4.0 s/5.5 s) were pseudo-randomly arranged (14 different orders), with the order of picture presentation randomized within each category.

Psychophysiological responses were recorded by using a 16-channel amplifier (Twente Medical Systems International [TMSi], EJ Oldenzaal, The Netherlands) and the recording software package Polybench 1.30 (TMSi) with a sampling frequency of 1024 Hz. Facial muscle activity was quantified using three pairs of Ag/AgCl electrodes (diameter: 2 mm), which were filled with EMG gel and placed onto the cleansed skin on the left side of the face.

After the passive picture viewing task was completed, participants rated all pictures presented in the same order on three dimensions using visual analogue scales. Participants were instructed to view each picture as long as desired and were asked to rate each picture regarding valence (*pleasant* [1] to *unpleasant* [9]), arousal (*relaxed* [1] to *aroused* [9]), and craving (*not at all* [1] to *very strongly* [9]).

Appendix A.3.3 Functional MRI assessment

The smoking cue-reactivity paradigm [11] used in the current study was adapted from the alcohol cue-reactivity paradigm employed by Vollstädt-Klein et al. [12]. The same 40 smoking-related stimuli used in the AAT were presented. The control stimuli consisted of 40 neutral pictures (e.g., toothbrushes, boxes with pencils), which were matched according to shape and color.

The task comprised 16 blocks (eight blocks with five smoking-related pictures each, and eight blocks with five neutral pictures each). While the block order was

pseudorandomized (no more than two blocks of the same category were presented consecutively), stimuli were presented randomly across blocks. Each stimulus was presented for four seconds. Participants were instructed to passively view the pictures. After each block, participants rated their current desire to smoke (“I want to smoke now.”) using a visual analogue scale ranging from *strongly disagree* (0) to *totally agree* (100) within a 10 s time limit. For this purpose, participants used buttons to move a cursor to its appropriate position on the scale. The task lasted approximately 10 min. See Figure A.3.3.1 for an illustration of the task design.

Figure A.3.3.1

fMRI smoking cue-reactivity task design

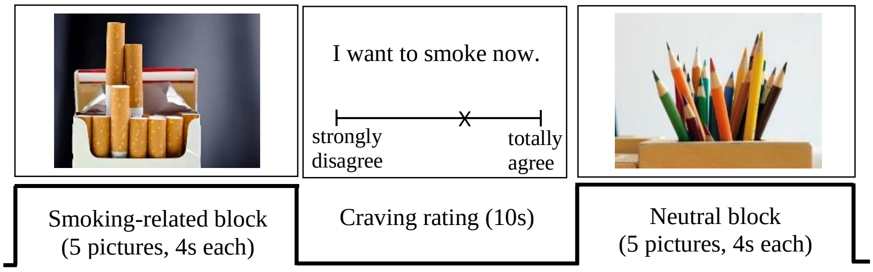

Note. fMRI smoking cue-reactivity task design with examples of smoking-related and neutral stimuli.

Neuroimaging data acquisition took place at the Neuroimaging Core Unit Munich (NICUM) of the LMU using a 3T Siemens Magnetom Prisma and a 32-channel head coil (Siemens AG, Erlangen, Germany). Functional sequences consisted of 320 volumes obtained using a T2*-weighted echo-planar imaging (EPI) sequence (48 slices per volume in ascending interleaved order with multiband factor 4, voxel size = 3 mm^3 isotropic, TR = 2000 ms, TE = 30 ms, flip angle = 45°, FoV = 210 mm). The first five functional volumes were discarded before preprocessing to account for T1 saturation effects. The high-resolution anatomical images consisted of 160 T1-weighted slices acquired using a magnetization-prepared rapid

gradient-echo (MP-RAGE) sequence (voxel size = 1 mm^3 isotropic, TR = 2300 ms, TE = 2.98 ms, flip angle = 9°, FoV = 256 mm).

Appendix A.4 Data preprocessing and measure extraction

The cognitive-behavioral and psychophysiological data were preprocessed according to the pre-defined procedure outlined in Wittekind et al. [5]. Due to the exclusion criteria for participants with excessive missing trials in each task, data for certain measures are unavailable for some participants. The final sample size for each measure is reported in Table 1.

Appendix A.4.1 Cognitive-behavioral tasks

Approach-Avoidance Task (AAT). For the analysis, only correctly executed trials were considered, meaning trials without any joystick movements in the wrong direction. Initial RTs (i.e., time between picture onset and first joystick response) less than 200 ms and greater than 2.5 *SD* above the group mean were excluded. Consistent with previous AAT studies [13], participants with more than 35% missing trials were excluded from further analysis. The AAT effect score was calculated by subtracting the median final RT (i.e., time between picture onset and full extension of the joystick) between push and pull movements during trials of smoking-related stimuli (i.e., $push_{smoking} – pull_{smoking}$).

Single-Target Implicit-Association Test (ST-IAT). Following the procedure outlined by Karpinski and Steinman [14], any response times less than 350 ms were excluded from the analysis. Error responses were replaced with the individual block mean plus a penalty of 400 ms. Participants with more than 20% errors in a test block were excluded from further analysis. The ST-IAT effect scores were calculated by subtracting the mean of compatible test trials ([smoking + positive/approach]) from the mean of incompatible test trials ([smoking + negative/avoidance]) and dividing the result by the pooled *SD* of all correct responses across the compatible and incompatible test blocks.

Stroop Task. Response trials with RTs below 200 ms or exceeding 2.5 *SD* above the group mean were excluded, as were incorrect trials. In line with the AAT preprocessing procedure, participants with more than 35% missing trials were excluded from further analysis. The Stroop interference score was calculated by subtracting the mean RT of control trials from the mean RT of incongruent trials.

Appendix A.4.2 Psychophysiological assessment

We used ANSLAB version 2.6 [15] to preprocess the EMG data. Some aspects of the preprocessing deviated from the procedure outlined in the preregistered study protocol [5]. We transparently describe when and why we adapted specific preprocessing steps. The data were filtered using a 50 Hz notch filter and a 28 Hz highpass filter, resulting in preprocessed data ranging from 28 to approximately 500 Hz. The data were then rectified. Contrary to the preregistration, an additional 500 Hz lowpass filter was not applied, as the prior preprocessing steps effectively reduced noise and extracted relevant EMG signals, making further filtering unnecessary. For the corrugator and zygomaticus muscle signal, EMG data were smoothed using ANSLAB's default moving average window of 50 ms. The 150 ms window stated in the preregistration was incorrect, as such a large window would have resulted in excessive smoothing and potential loss of significant signal information. For the startle response signal, a 15.9 Hz lowpass filter was applied, replacing the preregistered 50 ms moving average filter. This adjustment ensured that the preprocessing procedure for the startle response signal aligned with the recommendations of Blumenthal et al. [16].

Trials with evident movement artifacts or signal loss were marked as missing. In line with the preprocessing procedure for cognitive-behavioral measures, participants with more than 35% missing trials were excluded from the analyses. Table 1 presents the final sample size for each psychophysiological measure. Facial muscle activity of the corrugator (EMGcor) and zygomaticus (EMGzyg) muscles was assessed by calculating the average activity during a 7 s interval for trials with smoking-related and neutral stimuli, adjusted by subtracting the 1 s

baseline activity prior to stimulus onset. EMGcor and EMGzyg effect scores were then calculated by subtracting the mean facial muscle activity during neutral trials from that during smoking-related trials.

The startle response was scored by two independent raters, blinded to the picture category, with excellent inter-rater reliability (Cohen's kappa = .84). Disagreements were resolved through discussion to ensure consistency. For training purposes, a random selection of ten participants was rated collectively. Following the procedure recommended by Blumenthal et al. [16], invalid-rated trials (e.g., where the peak of activity did not occur within the predefined time window [20–200 ms] after probe onset) were excluded, and non-response trials (amplitude < 5 µV) were set to zero but included in the mean calculation (termed as magnitude). Participants with more than 35% missing trials (invalid-rated or non-response) were excluded. In contrast to previous studies (e.g., [7]), we did not screen participants for the presence of an appropriate startle response prior to participation, leading to the exclusion of a significant number of participants due to a high rate of non-response in the startle trials. EMGstartle effect scores were calculated by subtracting the mean magnitude of neutral trials from that of smoking-related trials.

Appendix A.4.3 Functional MRI assessment

Neuroimaging data analysis was performed with SPM12 (https://www.fil.ion.ucl.ac.uk/spm/software/spm12/) and MATLAB R2023a (The Mathworks, Natick, MA, USA). The SPM12 preprocessing pipeline was utilized with standard settings, including spatial realignment, co-registration, normalization to a standard 2 mm MNI template, and spatial smoothing with a Gaussian kernel (8 mm FWHM). Given the task block design, slice-timing correction was not performed. During preprocessing, four participants were excluded due to excessive head movement (> 3 mm in the x, y, or z direction or > 3° rotation in any direction), resulting in a final fMRI sample size of n = 113.

Statistical analysis of the preprocessed fMRI data at the first (individual) level was conducted using a general linear model (GLM). The model included one regressor for each of the three block conditions (smoking-related stimuli blocks, neutral stimuli blocks, and rating blocks). Regressors were modeled as boxcar functions and convolved with the canonical hemodynamic response function. A high-pass filter with a cut-off frequency of 1/128 Hz was used. Additionally, a constant term and motion correction parameters were included as regressors of no interest.

For the ROI analysis, we selected (sub)cortical grey matter regions associated with smoking cue-reactivity [smoking>neutral] in individuals who smoke, based on Lin et al.'s [17] meta-analysis. Masks were generated using the Automated Anatomical Labeling atlas (AAL3; [18]), encompassing the following five areas: (1) left anterior cingulate and paracingulate gyri (ACC; AAL3 labels: ACC_sub, ACC_pre, ACC_sup), (2) left angular gyrus (AAL3 labels: Angular), (3) right thalamus (AAL3 labels: Thal_AV, Thal_LP, Thal_VA, Thal_VL, Thal_VPL, Thal_IL, Thal_RE, Thal_MDm, Thal_MDl, Thal_LGN, Thal_MGN, Thal_PuA, Thal_PuM, Thal_PuL, Thal_PuI), and (4) right striatum (AAL3 labels: Caudate, N_Acc, Putamen, Olfactory). For the whole-brain analysis, regional labels for significant clusters were also derived from the AAL3 atlas. Mean β-estimates for the smoking and neutral conditions in the seven identified regions relevant for smoking cue-reactivity were extracted for statistical analyses.

Appendix B: Statistical analysis

Figure B.1

Model structure of the confirmatory factor analysis across all measures

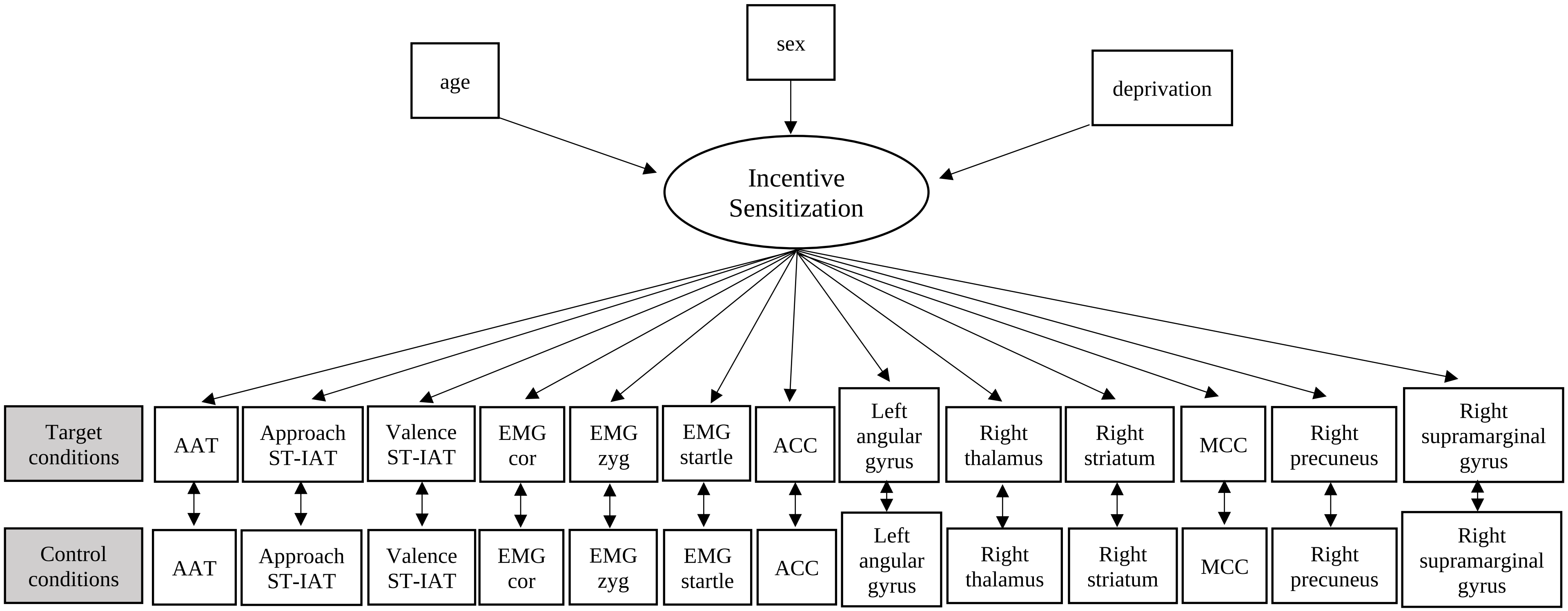

Note. AAT = Approach-Avoidance Task; ST-IAT = Single-Target Implicit-Association Test; EMGcor = Electromyography over the corrugator supercilii muscle; EMGzyg = Electromyography over the zygomaticus major muscle; EMGstartle = Electromyography over the orbicularis oculi muscle; ACC = left anterior cingulate and paracingulate gyri; MCC = right middle cingulate and paracingulate gyri.

Appendix C: Corona pandemic specifics

Assessment sessions were conducted individually, and outside of strict lockdown periods in Bavaria (Germany), two individuals wearing masks were permitted in the test room. Participants were allowed to remove their masks during testing, as the assessor left the room and monitored the session via video. Thus, sessions before and during the COVID-19 pandemic were highly comparable. However, from February to the end of May 2021, physiological assessments could not be conducted due to strict contact restrictions, affecting 46 participants (see Table 1 for the final n of each measure).

Although assessment sessions remained largely comparable, it remains conceivable that social restrictions during the COVID-19 pandemic influenced the results of our analyses. Lockdowns, regulatory measures, and related lifestyle changes may have affected smoking behavior, appetitive responses toward smoking-related stimuli, and inhibitory control. To account for this, we examined whether pandemic-related conditions had an impact on our data. For this purpose, we created a dummy-coded variable distinguishing between pre-COVID (0), low-to-moderate COVID conditions (1), and high COVID conditions (2), with the latter defined as periods when a state of disaster was officially declared in Bavaria. Such declarations occurred from March 16 to June 16, 2020 [19]; from December 9, 2020 to June 6, 2021 [20]; and from November 11, 2021 to May 11, 2022 [21]. This variable was included as a control in the regression models for hypothesis 2, but the results on the associations between appetitive responses, inhibitory control, and smoking-related variables as depicted in Table 3 remained unchanged.

Appendix D: Results of partial correlations

Partial correlation analyses revealed no significant associations between measures from different assessment methods, except that greater zygomaticus activity was significantly associated with higher right supramarginal gyrus activity during exposure to smoking-related stimuli (non-significant after FDR-correction; see Table C.1). Several significant intercorrelations were observed between measures within the cognitive-behavioral and neural assessment methods. Among cognitive-behavioral measures, faster RTs in compatible trials of the approach ST-IAT (smoking–approach) were significantly correlated with faster RTs in compatible trials of the valence ST-IAT (smoking–positive), and with faster pull movements during smoking-related trials in the AAT (all effects non-significant when using AAT and ST-IAT difference scores as outcome measures). Most neural measures were significantly correlated, except for MCC and right precuneus activity with ACC and left angular gyrus activity. No significant intercorrelations emerged among EMG measures.

Table D.1

Partial correlations between measures

Measures	**Cognitive-behavioral**				**Psychophysiological**			**Neural**						
	AAT	Approach ST-IAT	Valence ST-IAT	Stroop	EMG cor	EMG zyg	EMG startle	ACC	Left angular gyrus	Right thalamus	Right striatum	MCC	Right precuneus	Right supramarginal gyrus
**Cognitive-behavioral**														
AAT	1													
Approach ST-IAT	**.107 (.048)**[b]	1												
Valence ST-IAT	.078 (.150)	**.324 (<.001)**[a,c]	1											
Stroop	.064 (.235)	.001 (.984)	-.042 (.444)	1										
**Psychophysiological**														
EMGcor	-.024 (.686)	.020 (.741)	.166 (.051)	.011 (.847)	1									
EMGzyg	.070 (.233)	.007 (.912)	-.010 (.869)	-.003 (.965)	-.075 (.206)	1								
EMGstartle	.044 (.572)	-.081 (.293)	-.030 (.699)	.035 (.654)	-.126 (.103)	.124 (.106)	1							
**Neural**														
ACC	-.014 (.885)	.052 (.594)	.064 (.512)	.122 (.213)	-.031 (.760)	.131 (.187)	.174 (.187)	1						
Left angular gyrus	.164 (.092)	.054 (.578)	.007 (.939)	.124 (.206)	.015 (.879)	.101 (.312)	.141 (.286)	**.600 (<.001)**[a]	1					
Right thalamus	.019 (.843)	-.041 (.673)	.065 (.500)	-.003 (.975)	.084 (.406)	.124 (.213)	.108 (.416)	**.568 (<.001)**[a]	**.466 (<.001)**[a]	1				
Right striatum	.163 (.093)	-.046 (.637)	-.015 (.877)	-.027 (.781)	.004 (.972)	.112 (.260)	.175 (.184)	**.511 (<.001)**[a]	**.469 (<.001)**[a]	**.702 (<.001)**[a]	1			
MCC	.121 (.215)	-.172 (.076)	.095 (.325)	-.032 (.742)	.183 (.067)	.031 (.759)	-.223 (.090)	.140 (.147)	.166 (.085)	**.515 (<.001)**[a]	**.436 (<.001)**[a]	1		
Right precuneus	.026 (.787)	-.125 (.197)	.048 (.620)	.040 (.683)	.039 (.699)	.025 (.803)	-.113 (.392)	.034 (.724)	.031 (.746)	**.483 (<.001)**[a]	**.323 (<.001)**[a]	**.494 (<.001)**[a]	1	
Right supramarginal gyrus	.113 (.247)	-.018 (.850)	.034 (.725)	.054 (.580)	.070 (.486)	**.205 (.038)**	.231 (.078)	**.638 (<.001)**[a]	**.643 (<.001)**[a]	**.649 (<.001)**[a]	**.554 (<.001)**[a]	**.447 (<.001)**[a]	**.234 (.014)**	1

Note. Non-parametric partial correlation coefficients (Spearman's rho), controlled for age, sex, and deprivation (if applicable), are reported with

corresponding *p*-values in parentheses. Significant *p*-values are indicated in bold. AAT = Approach-Avoidance Task; ST-IAT = Single-Target Implicit-Association Test; EMGcor = Electromyography over the corrugator supercilii muscle; EMGzyg = Electromyography over the zygomaticus major muscle; EMGstartle = Electromyography over the orbicularis oculi muscle; ACC = left anterior cingulate and paracingulate gyri; MCC = right middle cingulate and paracingulate gyri.

[a] *p*-value remains significant after Benjamini-Hochberg correction.

[b] Using the AAT and approach ST-IAT difference scores, no significant correlation emerged (r = -.009, p = .864).

[c] Using the approach and valence ST-IAT difference scores, no significant correlation emerged (r = .054, p = .312).

References

1. Rinck M, Becker ES. Approach and avoidance in fear of spiders. *J Behav Ther Exp Psychiatry*. 2007;38(2):105-120. https://doi.org/10.1016/j.jbtep.2006.10.001.
2. Khazaal Y, Zullino D, Billieux J. The Geneva Smoking Pictures: development and preliminary validation. *Eur Addict Res*. 2012;18(3):103-109. https://doi.org/10.1159/000335083.
3. Oliver JA, Drobes DJ. Visual search and attentional bias for smoking cues: the role of familiarity. *Exp Clin Psychopharmacol*. 2012;20(6):489-496. https://doi.org/10.1037/a0029519.
4. Wigboldus DHJ, Holland RW, van Knippenberg A. *Single target implicit associations:* [Unpublished manuscript]; 2004.
5. Wittekind CE, Takano K, Sckopke P, et al. Efficacy of approach bias modification as an add-on to smoking cessation treatment: study protocol for a randomized-controlled double-blind trial. *Trials*. 2022;23(1):223. https://doi.org/10.1186/s13063-022-06155-6.
6. Stroop JR. Studies of interference in serial verbal reactions. *J Exp Psychol*. 1935;18(6):643-662. https://doi.org/10.1037/h0054651.
7. Geier A, Mucha RF, Pauli P. Appetitive nature of drug cues confirmed with physiological measures in a model using pictures of smoking. *Psychopharmacology (Berl)*. 2000;150(3):283-291. https://doi.org/10.1007/s002130000404.
8. Mucha RF, Pauli P, Weber M, Winkler M. Smoking stimuli from the terminal phase of cigarette consumption may not be cues for smoking in healthy smokers. *Psychopharmacology (Berl)*. 2008;201(1):81-95. https://doi.org/10.1007/s00213-008-1249-x.
9. Stippekohl B, Winkler M, Mucha RF, et al. Neural responses to BEGIN- and END-stimuli of the smoking ritual in nonsmokers, nondeprived smokers, and deprived smokers. *Neuropsychopharmacol*. 2010;35(5):1209-1225. https://doi.org/10.1038/npp.2009.227.

10. Lang PJ, Bradley MM, Cuthbert BN. *International affective picture system (IAPS): Affective ratings of pictures and instruction manual. Technical Report A-8.* Gainesville: The Center for Research in Psychophysiology, University of Florida; 2008.
11. Ekhtiari H, Zare-Bidoky M, Sangchooli A, et al. A methodological checklist for fMRI drug cue reactivity studies: development and expert consensus. *Nat Protoc*. 2022;17(3):567-595. https://doi.org/10.1038/s41596-021-00649-4.
12. Vollstädt-Klein S, Loeber S, Kirsch M, et al. Effects of cue-exposure treatment on neural cue reactivity in alcohol dependence: a randomized trial. *Biol Psychiatry*. 2011;69(11):1060-1066. https://doi.org/10.1016/j.biopsych.2010.12.016.
13. Wiers RW, Eberl C, Rinck M, Becker ES, Lindenmeyer J. Retraining automatic action tendencies changes alcoholic patients' approach bias for alcohol and improves treatment outcome. *Psychol Sci*. 2011;22(4):490-497. https://doi.org/10.1177/0956797611400615.
14. Karpinski A, Steinman RB. The single category implicit association test as a measure of implicit social cognition. *J Pers Soc Psychol*. 2006;91(1):16-32. https://doi.org/10.1037/0022-3514.91.1.16.
15. Blechert J, Peyk P, Liedlgruber M, Wilhelm FH. ANSLAB: Integrated multichannel peripheral biosignal processing in psychophysiological science. *Behav Res Methods*. 2016;48(4):1528-1545. https://doi.org/10.3758/s13428-015-0665-1.
16. Blumenthal TD, Cuthbert BN, Filion DL, Hackley S, Lipp OV, van Boxtel A. Committee report: Guidelines for human startle eyeblink electromyographic studies. *Psychophysiology*. 2005;42(1):1-15. https://doi.org/10.1111/j.1469-8986.2005.00271.x.
17. Lin X, Deng J, Le Shi, et al. Neural substrates of smoking and reward cue reactivity in smokers: a meta-analysis of fMRI studies. *Transl Psychiatry*. 2020;10(1):97. https://doi.org/10.1038/s41398-020-0775-0.
18. Rolls ET, Huang C-C, Lin C-P, Feng J, Joliot M. Automated anatomical labelling atlas 3. *Neuroimage*. 2020;206:116189. https://doi.org/10.1016/j.neuroimage.2019.116189.

19. Bayerische Staatskanzlei. Bericht aus der Kabinettssitzung vom 16. Juni 2020 – Bayerisches Landesportal. https://www.bayern.de/bericht-aus-der-kabinettssitzung-vom-16-juni-2020/. Accessed August 23, 2025.
20. Bayerische Staatskanzlei. BayMBl. 2021 Nr. 383 - Verkündungsplattform Bayern. https://www.verkuendung-bayern.de/baymbl/2021-383/. Accessed August 23, 2025.
21. Bayerische Staatskanzlei. BayMBl. 2022 Nr. 287 - Verkündungsplattform Bayern. https://www.verkuendung-bayern.de/baymbl/2022-287/. Accessed August 23, 2025.
